# Trifluoperazine, a novel autophagy inhibitor, increases radiosensitivity in glioblastoma by impairing homologous recombination

**DOI:** 10.1186/s13046-017-0588-z

**Published:** 2017-09-05

**Authors:** Xin Zhang, Ran Xu, Chao Zhang, Yangyang Xu, Mingzhi Han, Bin Huang, Anjing Chen, Chen Qiu, Frits Thorsen, Lars Prestegarden, Rolf Bjerkvig, Jian Wang, Xingang Li

**Affiliations:** 1 Department of Neurosurgery, Qilu Hospital of Shandong University and Brain Science Research Institute, Shandong University, Jinan, 250012 People’s Republic of China; 2 Department of Radiation Oncology, Qilu Hospital of Shandong University, Jinan, 250012 People’s Republic of China; 3 Kristian Gerhard Jebsen Brain Tumour Research Centre, Department of Biomedicine, University of Bergen, 5009 Bergen, Norway; 4 The Molecular Imaging Center, Department of Biomedicine, University of Bergen, 5009 Bergen, Norway; 5 Department of Dermatology, Haukeland University Hospital, 5009 Bergen, Norway; 6 Department of Oncology, Luxembourg Institute of Health, L-1526 Strassen, Luxembourg

**Keywords:** Trifluoperazine, Autophagy inhibitor, Radiosensitivity, Glioblastoma, Homologous recombination

## Abstract

**Background:**

Resistance to adjuvant radiotherapy is a major cause of treatment failure in patients with glioblastoma (GBM). Autophagy inhibitors have been shown to enhance the efficacy of radiotherapy for certain solid tumors. However, current inhibitors do not penetrate the blood-brain-barrier (BBB). Here, we assessed the radiosensitivity effects of the antipsychotic drug trifluoperazine (TFP) on GBM in vitro and in vivo*.*

**Methods:**

U251 and U87 GBM cell lines as well as GBM cells from a primary human biopsy (P3), were used in vitro and in vivo to evaluate the efficacy of TFP treatment. Viability and cytotoxicity was evaluated by CCK-8 and clonogenic formation assays. Molecular studies using immunohistochemistry, western blots, immunofluorescence and qPCR were used to gain mechanistic insight into the biological activity of TFP. Preclinical therapeutic efficacy was evaluated in orthotopic xenograft mouse models.

**Results:**

IC50 values of U251, U87 and P3 cells treated with TFP were 16, 15 and 15.5 μM, respectively. TFP increased the expression of LC3B-II and p62, indicating a potential disruption of autophagy flux. These results were further substantiated by a decreased Lysotracker Red uptake, indicating impaired acidification of the lysosomes. We show that TFP and radiation had an additive effect when combined. This effect was in part due to impaired TFP-induced homologous recombination. Mechanistically we show that down-regulation of cathepsin L might explain the radiosensitivity effect of TFP. Finally, combining TFP and radiation resulted in a significant antitumor effect in orthotopic GBM xenograft models.

**Conclusions:**

This study provides a strong rationale for further clinical studies exploring the combination therapy of TFP and radiation to treat GBM patients.

## Background

Glioblastoma (GBM) is the most aggressive of all intracranial tumors. Despite multimodal treatment including surgical resection, chemotherapy and radiotherapy, the median survival is only 14.6 months [1, 2]. Radiotherapy targets cancer cells by causing DNA damage and is a highly cost-effective treatment [3]. However, DNA damage induced by radiation triggers a series of signaling cascades promoting cell survival, including DNA repair, cell cycle arrest, and autophagy, all of which mediate radioresistance and prevent further clinical efficacy [4].

Autophagy is a lysosome-dependent degradation and cell survival process which represents a therapeutic target in cancer treatment due to its role in DNA damage [5]. Several studies have shown that inhibiting autophagy could increase the radiosensitivity of tumor cells [6–8]. Currently, multiple clinical trials have been initiated that combine conventional anti-cancer therapies with inhibition of autophagy [9–11]. Previous studies have shown that Bafilomycin A1, a vacuolar H^+^-ATPase inhibitor, increases DNA degradation and significantly increases survival after irradiation of MCF-7 (human breast adenocarcinoma), LoVo (human colon adenocarcinoma), and LNCaP (human prostate carcinoma) cells [12]. However, due to the blood-brain-barrier (BBB), most autophagy inhibitors will not effectively benefit GBM patients. Therefore, identifying new autophagy inhibitors with improved pharmacokinetics for diseases in the central nervous system (CNS) is urgently needed.

Trifluoperazine (TFP) is a typical antipsychotic compound of the phenothiazine chemical class. It has been used in the treatment of schizophrenia for more than 50 years and relieves agitation in patients with behavioral problems, severe nausea and vomiting as well as severe anxiety [13]. Recently, an increasing number of studies have found that TFP has potent anti-tumor effects in lung cancer, malignant peripheral nerve sheath tumors and leukemia [14–16]. Here, we examined the responses of GBM cells to TFP in vitro and in vivo and show that TFP inhibits autophagy by interfering with lysosome acidification. Moreover, TFP treatment impairs DNA damage repair following radiotherapy, providing a rationale for combining TFP with radiation therapy in GBMs.

## Methods

### Cell lines

Human glioma cell lines U87 and U251 were purchased from the cell bank of the Chinese Academy of Sciences and were cultured in Dulbecco’s modified Eagle’s medium (ThermoFisher Scientific; Waltham, MA, USA) containing 10% fetal bovine serum (ThermoFisher Scientific), glutamine (4 mM), penicillin (10 U/mL), and streptomycin (100 mg/mL). Normal human astrocytes (NHA) were purchased from Lonza (Walkersville, MD, USA) and were cultured in Astrocyte Medium BulletKit (Lonza) according to the manufacturer’s instructions. P3 is a primary GBM cell line isolated from a patient biopsy. The P3 tumor has the following molecular characteristics (+ [Chr 7, Chr19, 20q], −[1q42-q43, Chr9, Chr10, 20p] --[PIK3R1, CDKN2A/B]. P3 cells were cultured in Neurobasal Medium (ThermoFisher Scientific) containing penicillin (10 U/mL), streptomycin (100 mg/mL), B27 supplement (20 μL/mL), FGF (20 ng/mL), EGF (20 ng/mL) and heparin (32 IE/mL).

### Cell viability assay

The cytotoxic effect of TFP (Sigma; St. Louis, MO, USA) on the GBM cell lines, P3 and NHA cells was determined using the CCK-8 assay (Dojindo; Kumamoto, Japan). Cells were suspended in DMEM with 10% fetal bovine serum (FBS) or Neurobasal Medium (for P3) and seeded into 96-well, flat-bottomed plates (5 × 10^3^ cells/well). After incubation overnight at 37 °C, cells were pretreated with PBS or TFP (0–30 μM). After 24 h or 48 h of culture, cells were incubated for an additional 2 h at 37 °C with 100 μL of serum-free DMEM or Neurobasal Medium (for P3 cells) containing 10 μL of CCK-8, and absorbance was measured at a wavelength of 450 nm using a microplate reader (BioRad; Hercules, CA, USA).

### EdU proliferation assay

The tumor cells (2.5 × 10^4^ cells/well) were seeded into 24-well, flat-bottomed plates. After 24 h, cells were treated with PBS, 5 and 10 μM of TFP for an additional 48 h in DMEM with 10% serum, and subsequently stained with EdU using the Apollo Detection Kit (Ribobio; Guangzhou, China) according to the manufacturer’s instructions. EdU positive cells were counted from at least 10 random fields by fluorescence microscopy (Leica DMi8; Leica Microsystems, Wetzlar, Germany).

### Western blot analysis

After treatment of different doses of TFP, 100 nM bafilomycin A1, 2.5 μM rapamycin, 4 Gy radiation at a dose rate of 1.8 Gy/min using a linear accelerator (Primus Hi; Siemens Medical Instruments; Erlangen, Germany) or 5 μM TFP for 24 h before receiving one dose of 4 Gy, whole-cell protein extracts (20-50 mg) were prepared using a radioimmunoprecipitation assay buffer (RIPA; Thermo Fisher Scientific) supplemented with protease inhibitor cocktail (Cell Signaling Technology; Beverly, MA, USA). Proteins were resolved by sodium dodecyl sulfate-polyacrylamide gel electrophoresis and transferred to a polyvinylidene difluoride membrane. Membranes were blocked with 5% skimmed milk in Tris-buffered saline containing 0.1% Tween-20, and subsequently incubated with primary and indicated secondary antibodies (Thermo Fisher Scientific). Proteins on western blots where visualized using the Chemiluminescent Reagents Kit (Millipore, Billerica, MA, USA). Chemiluminescent signals were detected with the ChemiDoc XRS+ (Bio-Rad, Hercules, CA, USA) and quantified using Image Lab 3.0 software (Bio-Rad). The following primary antibodies were used for western blotting: beta-tubulin, LC3BI/II, phospho-histone H2A.X (Ser139; also known as γ-H2A.X), P62 and survivin (Cell Signaling Technology; Beverly, MA, USA); GAPDH, BRCA1, BRCA2 and Rad51 were purchased from Santa Cruz (Dallas, TX, USA).

### GFP-LC3 transient transfection

Cells were transiently transfected with the pSELECT-GFP-LC3 plasmid (Genepharma; Shanghai, China) using Lipofectamine 2000 reagent (ThermoFisher Scientific) according to the manufacturer’s instructions. After being treated with PBS or 10 μM TFP for 24 h, cells were observed using a Leica TCS SP5 Confocal Laser Scanning Microscope (Leica Microsystems) and GFP-LC3 puncta per cell was counted. Ten random fields were obtained per treatment group.

### Transmission electron microscopy

Cells were fixed in 3% glutaraldehyde in PBS for 2 h, washed five times with 0.1 M cacodylate buffer, and post-fixed with 1% OsO_4_ in 0.1 M cacodylate buffer containing 0.1% CaCl_2_ for 1.5 h at 4 °C. Cells were dehydrated in graded alcohol series and embedded in epoxy resin. Ultrathin sections were cut and stained with uranyl acetate and lead citrate. Images were obtained using a JEM-1200EX II electron microscope (JEOL, Tokyo, Japan).

### LysoTracker staining

Following treatment with PBS (control), 100 nM bafilomycin A1 and 10 μM TFP for 48 h, U251 and U87 cells were rinsed 3 times with fresh medium, and Lyso-Tracker Red (diluted in DMEM with 10% FBS; ThermoFisher Scientific) was added to a final concentration of 66 nM. Cells were incubated for 30 min at 37 °C and rinsed with phosphate-buffered saline (PBS). Nuclei were stained with 5 μg/ml Hoechst 33,342 (ThermoFisher Scientific), and live cells were observed using a Leica DMi8 fluorescence microscope.

### Comet assay (single cell gel electrophoresis assay)

Comet assays were performed according to a previously described protocol [17]. Briefly, after treatment with PBS, TFP, radiation or combination treatment, cells were thoroughly mixed with low melting point agarose solution. Radiation treatment was carried out with a single dose of 4 Gy at a dose rate of 1.8 Gy/min using a linear accelerator (Primus Hi). The cell suspension was spread on a Comet Slide (CometAssay® Kit, Trevigen; Gaithersburg, MD, USA) covered with 1.5% normal melting agarose. Slides were immersed in prepared lysis solution, treated with Tris-EDTA buffer (10 mM TrisCl, pH 7.5, 1 mM EDTA), and then placed horizontally on an electrophoresis tray filled with alkaline solution (300 mM NaOH, 1 mM EDTA). Electrophoresis was conducted at room temperature with an electrical field of 25 V and a current of 300 mA for 20 min. After electrophoresis, the slides were stained with GelRed (Biotium; Fremont, CA, USA). Slides were examined under fluorescence microscopy. Cells were analyzed using the Comet Assay Software Project (CASP). Olive tail moment (OTM) was used to quantify the extent of DNA damage.

### Immunofluorescence

Immunofluorescence detection of γ-H2A.X foci was performed to monitor formation of DNA double strand breaks (DSBs). Cells cultured on coverslips were treated with PBS or 5 μM of TFP before receiving one dose of 4 Gy at a dose rate of 1.8 Gy/min using a linear accelerator (Primus Hi). At indicated time points (2, 6, 12 and 24 h), the cells were rinsed with PBS and then fixed in 4% paraformaldehyde before permeabilisation with 0.3% Triton X-100. After blocking with 5% BSA (Sigma), cells were incubated with diluted primary antibody for γ-H2A.X overnight at 4 °C, followed by staining with Fluorescein (FITC)-conjugated goat anti-rabbit IgG (ThermoFisher Scientific). Finally, the samples were mounted in mounting medium containing DAPI (ThermoFisher Scientific). Three random fields were examined at a magnification of ×63 by a Leica TCS SP5 Confocal Laser Scanning Microscope.

### Colony formation assay

U251 and U87 cells (3 × 10^3^ cells/well) were plated in six-well plates. The adherent cells were then treated with PBS or 5 μM TFP for 24 h before receiving one dose of 4 Gy at a dose rate of 1.8 Gy/min whereupon they were incubated for 14 days. Then colonies were washed with PBS, fixed in 4% paraformaldehyde, stained with 0.1% crystal violet and counted. Colonies consisting of more than 50 cells were counted as surviving colonies.

### Apoptosis analysis

Apoptosis was measured by quantifying cleaved-caspase 3 and 7 activity using Cell Event Caspase 3/7 Green Detection Reagent (Life Technologies, Carlsbad, CA, USA). Cells were plated in 96-well plates with a density of 3000 cells per well and allowed to adhere. Thereafter, the cells were treated with PBS or 5 μM TFP for 24 h before receiving one dose of 4 Gy at a dose rate of 1.8 Gy/min in a linear accelerator (Primus Hi). Then the cells were inspected using an IncuCyte Zoom live cell imaging system (Essen BioScience, Ann Arbor, MI, USA).

### Homologous recombination (HR) assay

An HR assay (Norgen Biotek, Thorold, ON, Canada) was performed on U251 and U87 cells according to manufacturer’s instructions. Briefly, at day 3 after TFP treatment, cells were transfected with a positive control plasmid or two HR dl plasmids (dl-1 and dl-2). After 24 h of transfection, DNA was isolated using the Wizard genomic DNA purification kit (Promega, Madison, WI, USA). qPCR was performed with the supplied primers using a Roche LightCycler 480 II (Roche Applied Science, Indianapolis, IN, USA).

### Cathepsin B and L activity

Activity of cathepsin B and L in U251 and U87 cells was tested using a Fluorometric Assay Kit (Abcam, Cambridge, UK) according to the manufacturer instructions. Briefly, after treatment with PBS (control) or 5 μM TFP for 24 h, the cells were lysed and supernatants were incubated with cathepsin-B (Ac-RR-AFC) or L (AC-FR-AFC) substrates at 37 °C for 1.5 h. Then samples were measured on a fluorescent microplate reader at excitation/emission wavelength = 400/505 nm. After subtracting the background control (buffer) from sample readings, activity of cathepsin B and L was determined by comparing results from TFP treated cells with the level from controls.

### Transfection of siRNA

U251 and U87 cells were transfected with siRNA twice at a 24-h interval with lipofectamine 2000. The final concentration of siRNAs was 50 nM. Sequences for the siRNAs used were the following: cathepsin L, 5′-GATGCACAACAGATTATACTT-3′; nontargeting siRNA controls, 5′-UUCUCCGAACGUGUCACGUTT-3′ (Genepharma). Western blot analysis was used to assess the downregulation of cathepsin L.

### Intracranial implantation and drug therapy

All animal protocols were approved by the ethics committee at the Shandong University (Jinan, China) and conducted according to the national regulations in China. For implantations, nude mice were anesthetized with 4% chloral hydrate (300 mg/kg) and placed in a stereotactic frame. Using aseptic surgical procedures, an incision was made in the parietal scalp, and a small burr hole was drilled 2.5 mm lateral to the bregma. U251 and P3 cells (1 × 10^6^ cells/mouse) were implanted 2.0 mm into the right striatum using a Hamilton syringe (Hamilton Co., Reno, NV, USA). Two weeks later, mice were randomly divided into four groups (6 mice/group). Groups 1 and 2 were injected intraperitoneally (IP) with PBS or TFP (1 mg/kg, 5 days/week). Group 3 was given three doses of localized irradiation (5 Gy) at days 15, 20, and 25 after implantation following IP injection of PBS. Group 4 was irradiated three times following IP injection of TFP. Mice were sacrificed when central nervous system symptoms (such as poor ambulation, lethargy, hunched posture) or weight loss > 20% body mass developed. The mice were anesthetized with chlorohydrate and perfused transcardially with 4% paraformaldehyde in PBS. Whole brains were removed, post-fixed overnight in 4% paraformaldehyde in PBS, coronally sectioned into 5 slices, and paraffin embedded. Tissue sections were cut (10 μm) and incubated with primary antibodies as indicated. The following primary antibodies were used for immunohistochemistry: Ki67 (Abcam); γ-H2A.X (Cell Signaling Technology), and Rad51 (Santa Cruz).

### Statistical analysis

Unpaired T-tests were performed using SPSS software 13.0 (SPSS Inc., Chicago, IL). Results are presented as the mean ± SE. *P*-values <0.05 were considered statistically significant.

## Results

### TFP inhibits GBM cell growth in vitro

**Fig. 1:**
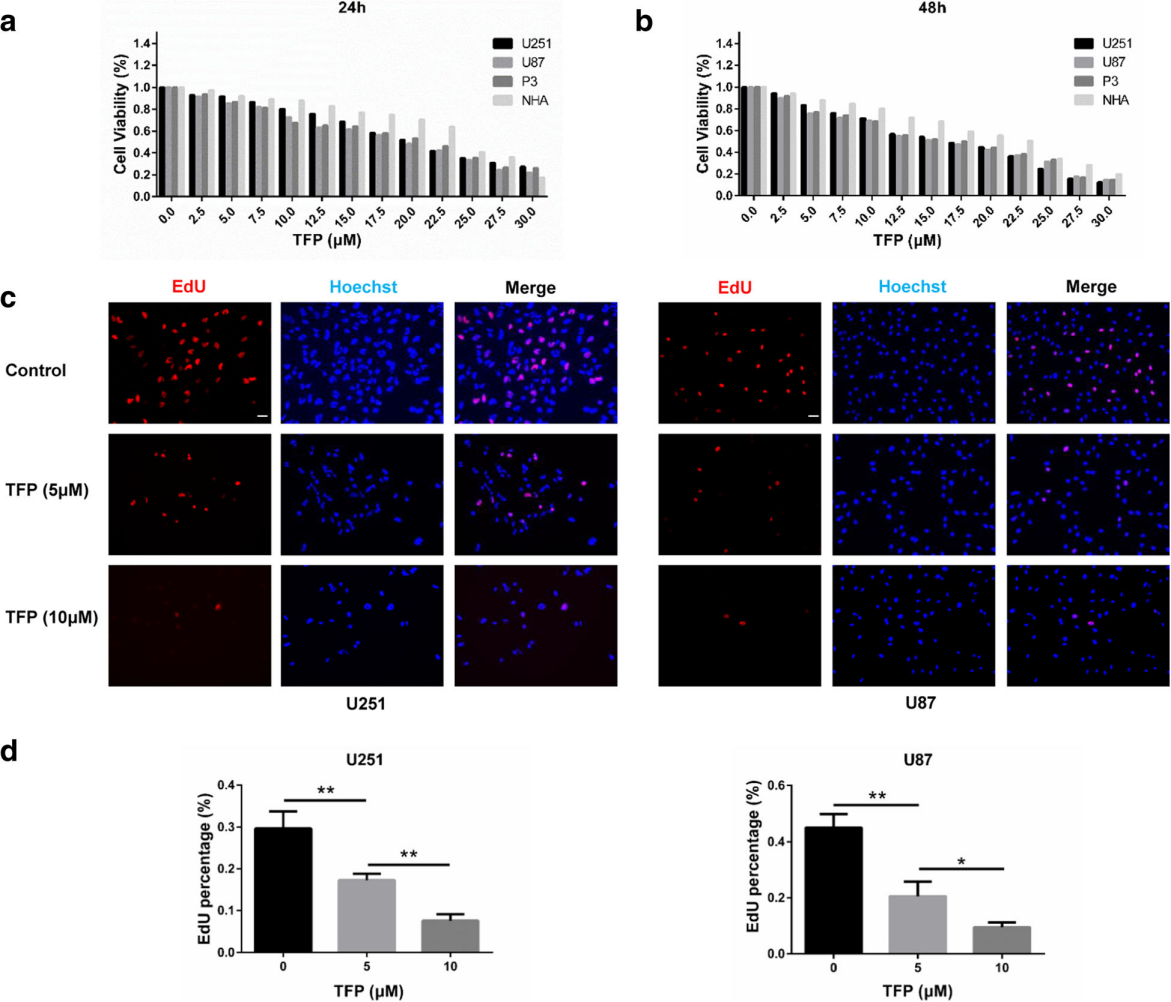
TFP inhibited GBM cell growth *in vitro*. **a** and **b** Cell viability of U251, U87, P3 and NHA as determined in CCK8 assays after treatment with TFP for 24 or 48 h. **c** Fluorescence microscopy of EdU assays after treatment of U251 and U87 cell lines with TFP at concentrations of 0, 5, and 10 μM. **d** Quantification of EdU assays. *P < 0.05; **P < 0.01; size bars = 50 μm

The cytotoxic effects of TFP on tumor cells in vitro were determined using the CCK-8 assay (Fig. 1a, b). The IC50 values of TFP for U251, U87 and P3 cells were 16 μM, 15 μM, 15.5 μM, respectively. GBM cells were significantly more sensitive to TFP compared to normal human astrocytes (IC50 22.5 μM, *P* < 0.05). These results were further substantiated by the use of a proliferation assay where EdU was incorporated in U251 and U87 cells. The number of EdU positive cells decreased in a dose dependent manner in both cell populations (Fig. 1c, d). These results were further confirmed by a reduced clonogenic ability and increased caspase activity following TFP treatment (Fig. 5c, d).

### TFP interferes with autophagy flux in GBM cells

**Fig. 2:**
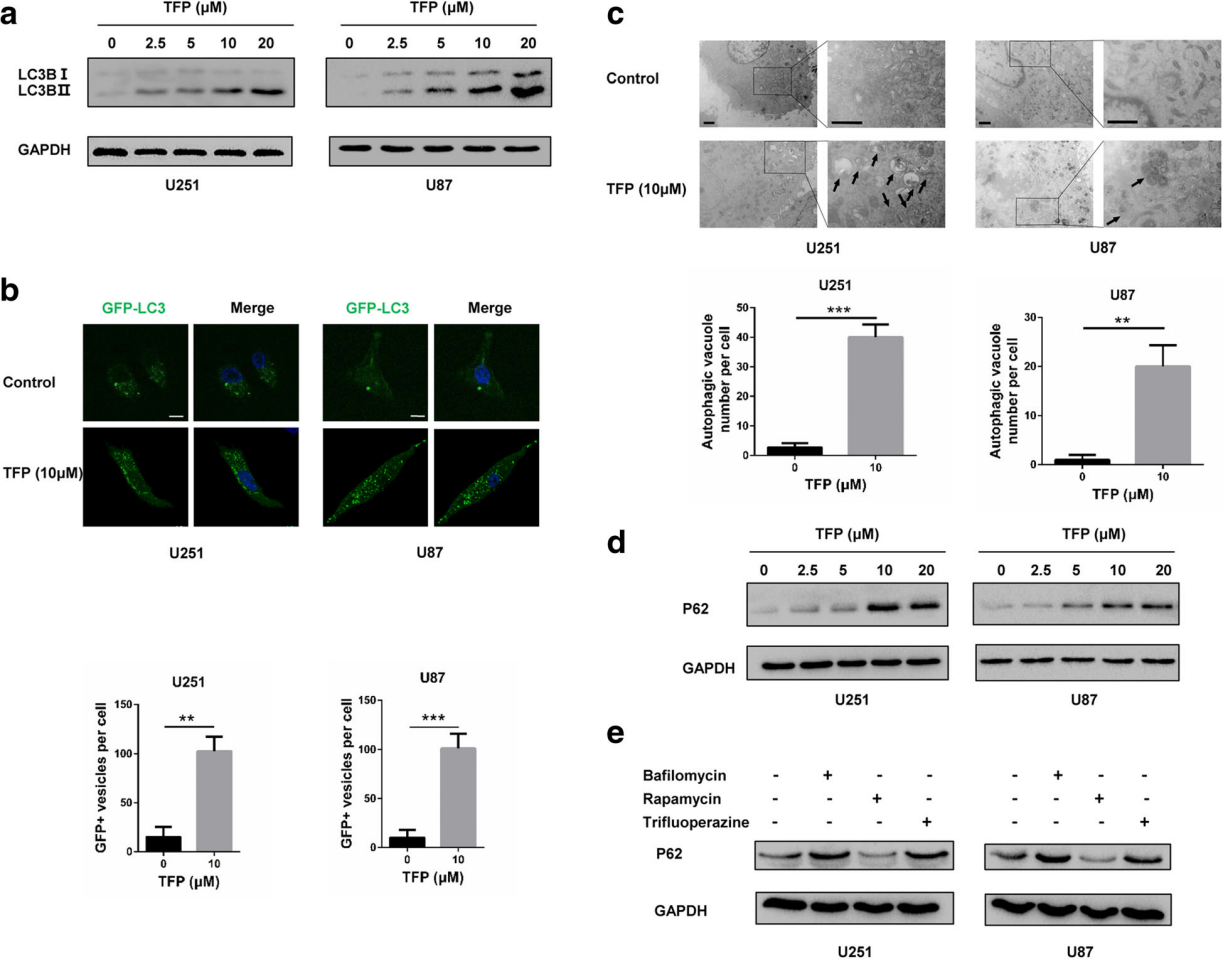
TFP interrupts autophagy flux of GBM cells. **a** Western blot showing expression of LC3BI/II in U251 and U87 cells incubated for 24 h with TFP at increasing concentrations. **b** Images of LC3 puncta in GBM cells transfected with pSELECT-GFP-LC3 and treated with PBS and 10 μM TFP for 48 h. **c** Images from transmission electron microscopy showing characteristic autophagic vacuoles (Black arrows) in GBM cells after treatment with PBS or 10 μM TFP for 48 h. **d** and **e** Western blot showing p62 levels in GBM cells after treatment with increasing concentrations of TFP, 100 nM bafilomycin A1, 2.5 μM rapamycin and 10 μM TFP for 48 h. **P < 0.01; ***P < 0.001; size bars in **b** = 10 μm, size bars in **c** = 1 μm

Previous studies have suggested that TFP induces autophagy [18]. LC3B, a membrane component of autophagosomes, is used as a marker for the induction of autophagy. Analyses of LC3B by western blot showed increased levels after upon TFP treatment (Fig. 2a). To visualize the development of autophagosomes following TFP treatment, we transiently transfected GBM cells with a GFP-LC3 expression construct. Confocal microscopy showed an increase in fluorescence puncta in TFP treated cells after 24 h (Fig. 2b). Transmission electron microscopy (TEM) represents the gold standard for detecting autophagic vacuoles (AVs). More AVs were detected in TFP treated cells compared to the control group (Black arrows, Fig. 2c).

Autophagy is a dynamic multistage process where the assembly of autophagosomes is an early event. The assembly of autophagosomes is therefore not directly connected to autophagosome degradation. Thus, an accumulation of AVs in cells can occur either as a consequence of an increased autophagosome formation or decreased degradation [19]. The levels of SQSTM/p62, one of the most important long-lived proteins critical for autophagy, accumulated in a dose-dependent manner in response to TFP (Fig. 2d). This indicates that TFP blocks autophagy.

Rapamycin, which induces autophagy by inhibition of mTOR [20], and Bafilomycin A1 (BAF), which blocks autophagy by selectively inhibiting H^+^-ATPase [21], were used as positive controls. Our results show that p62 levels decreased in rapamycin treated cells whereas it was clearly increased in cells treated with TFP or BAF (Fig. 2e).

**Fig. 3:**
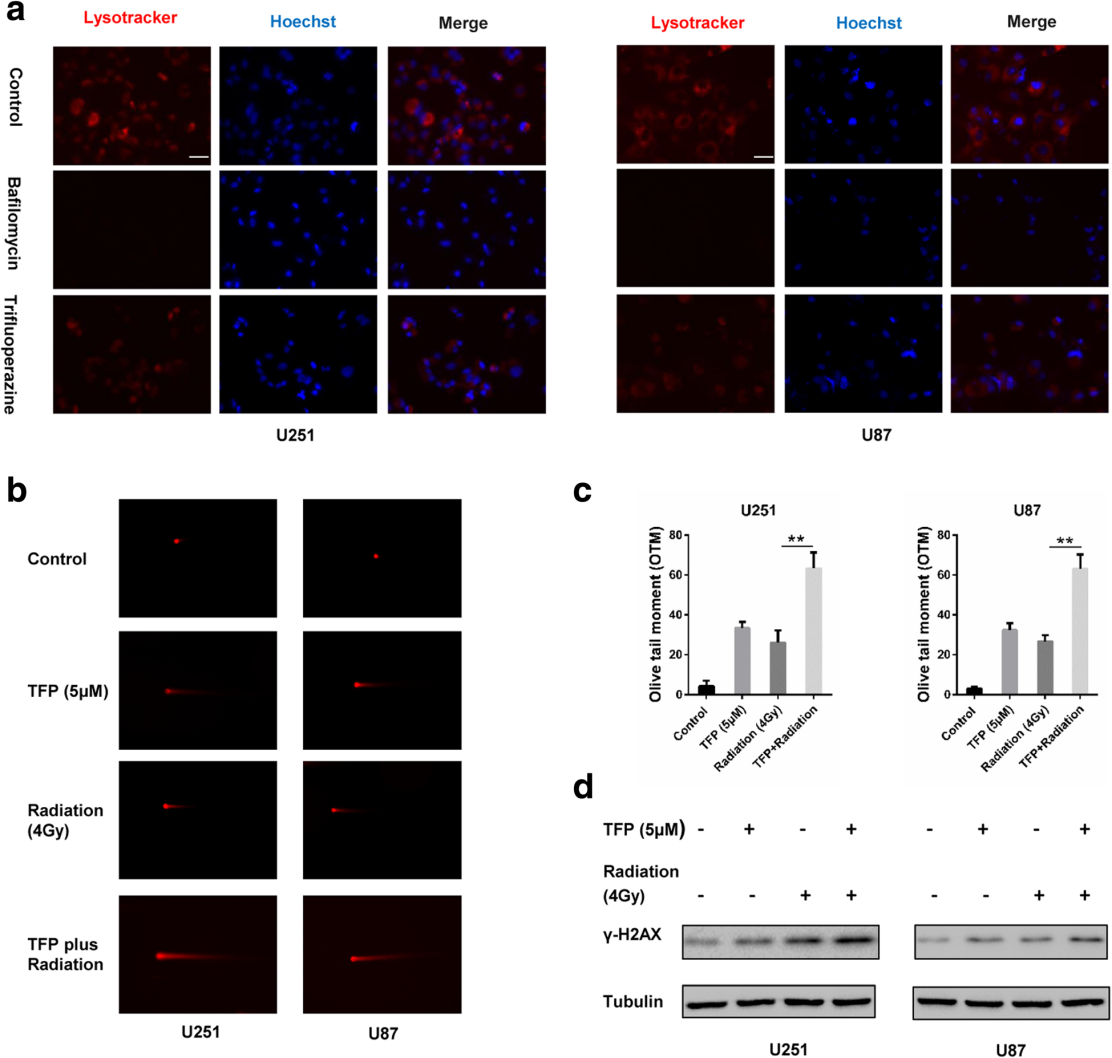
TFP influences the acidification of lysosomes and increases the radiosensitivity of GBM cells. **a** LysoTracker Red in cells after treatment with PBS, 100 nM bafilomycin A1 and 10 μM TFP for 48 h. Nuclei are stained with Hoechst. **b** and **c** Comet assays to evaluate DNA damage in cells after treatment with PBS, 5 μM TFP, or 4 Gy radiation for 48, or pretreated with 5 μM TFP for 24 h combined with 4 Gy radiation and TFP for another 24 h. **d** Western blot for γ-H2AX in GBM cells after treatment. **P < 0.01; size bars = 50 μm

BAF blocks autophagy flux by reducing the acidification of lysosomes. Altered lysosome function prevents fusion of autophagosomes with lysosomes. LysoTracker Red, which labels highly acidic lysosomal vacuoles and thus detects activity of vacuolar H^+^-ATPase (v-ATPase) [22], was used to determine the status of lysosomes after TFP or BAF treatment. Under fluorescence microscopy, lysosomes could not be detected with LysoTracker Red in TFP and BAF treated cells (Fig. 3a). These results indicate that TFP blocks autophagy by inhibiting the acidification of lysosomes.

### TFP enhances the radiosensitivity of GBM cells

A number of studies have demonstrated that inhibitors of autophagy enhance the sensitivity of cells to radiation therapy [6, 8, 23]. To investigate whether TFP affected radiosensitivity, we used the comet assay to measure DNA integrity in U251 and U87 cells. TFP or radiation treatment alone slightly increased the levels of DNA tails above controls, whereas combined treatment caused a significant increase in the appearance of DNA tails (Fig. 3b, c). Protein levels of γ-H2AX, a gold standard to detect the presence of DSBs [24], were correspondingly increased following combination treatment (Fig. 3d).

### TFP increases radiosensitivity by impairing homologous recombination repair

**Fig. 4:**
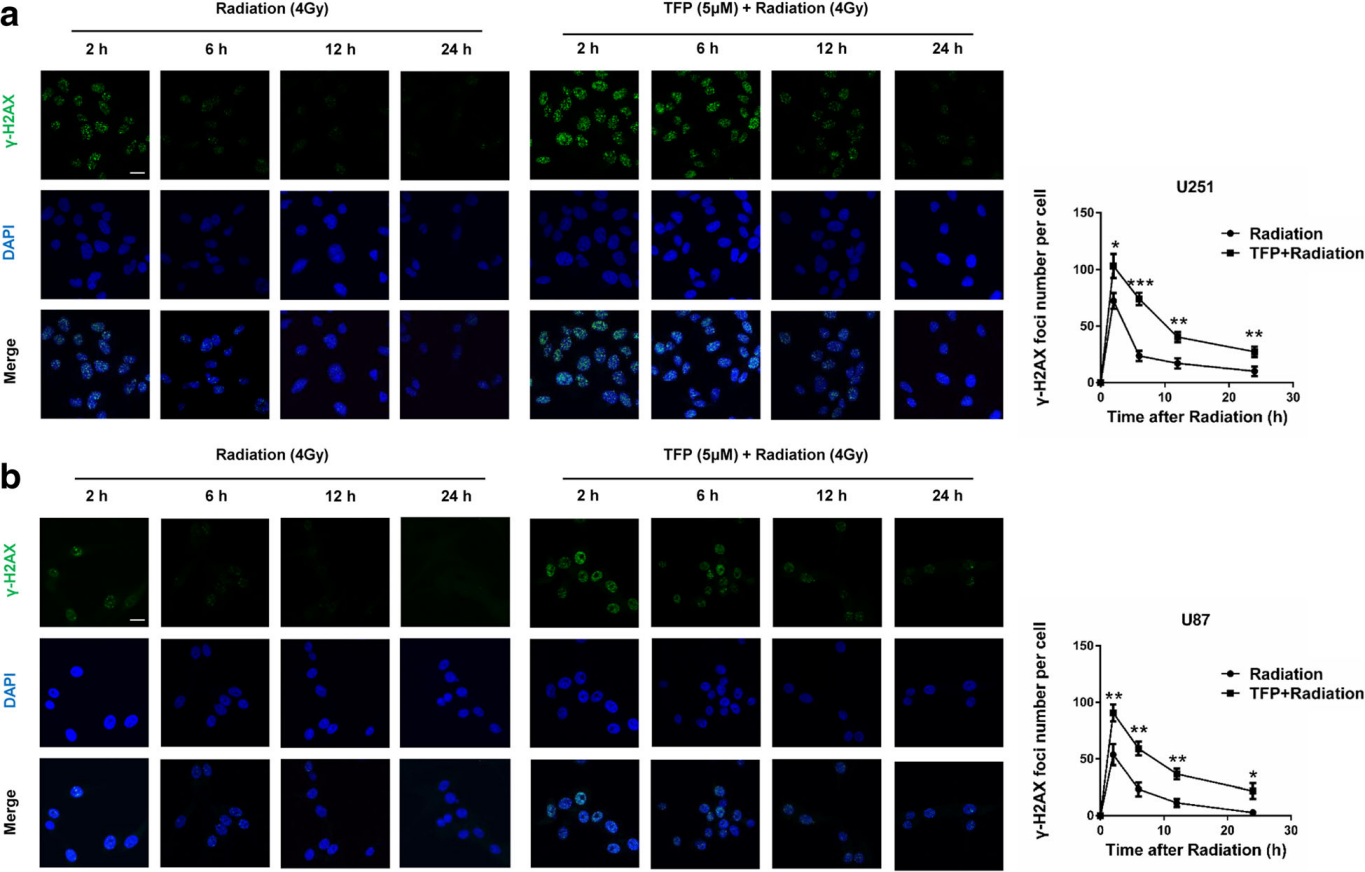
TFP decreases DNA damage repair in GBM cells. **a** and **b** Immunofluorescence for γ-H2AX at different time points after irradiation in GBM cells treated with PBS or TFP. *P < 0.05; **P < 0.01; ***P < 0.001; size bars = 10 μm

**Fig. 5:**
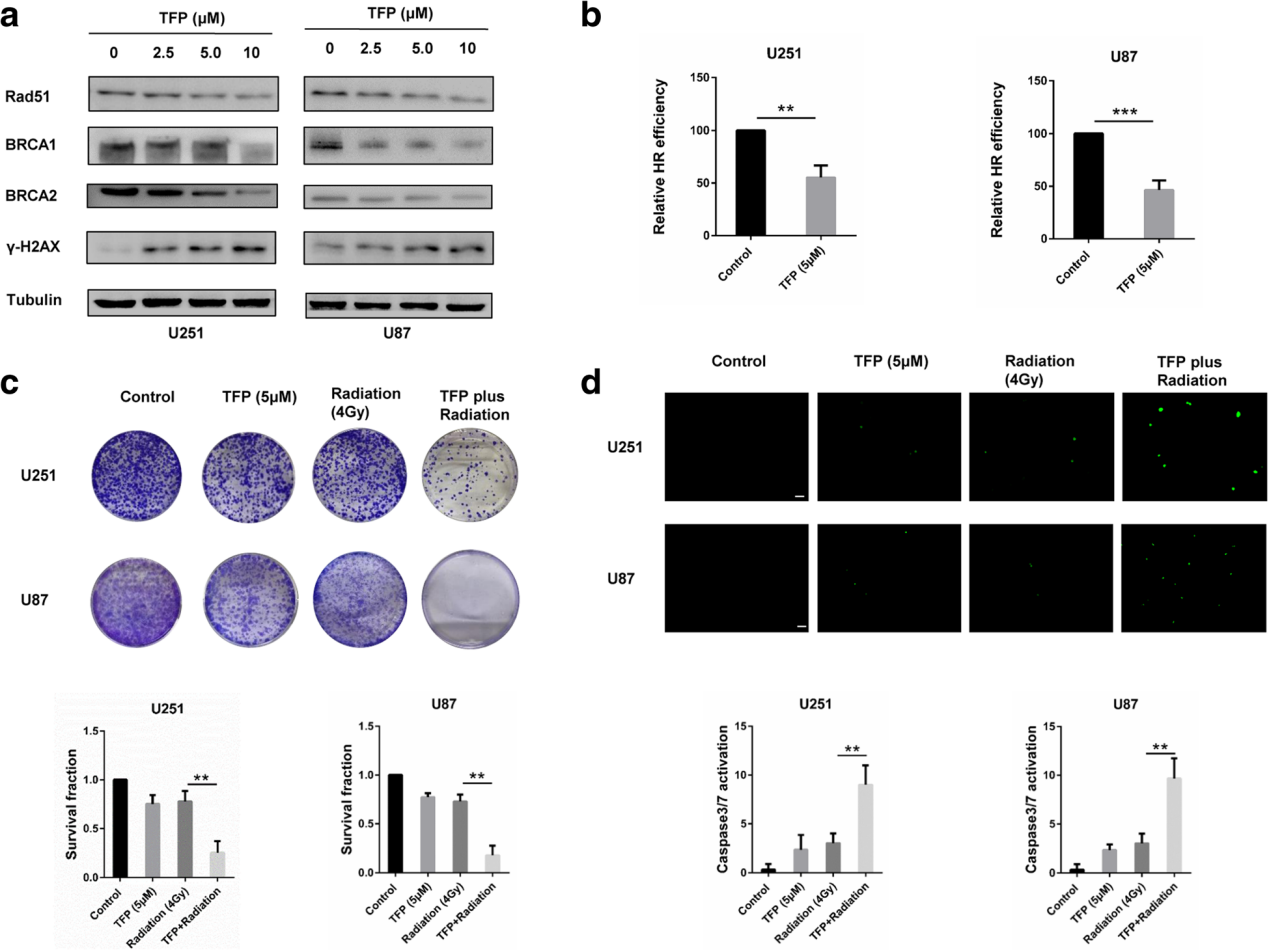
TFP decreases HR repair and causes synergistic anticancer effect with radiation in GBM cells. **a** Western blots for Rad51, BRCA1, BRCA2 and γ-H2AX in U251 and U87 cells after different doses of TFP treatment. **b** HR activity of U251 and U87 after treatment of PBS and 5 μM TFP. **c** Colony formation of U251 and U87 after treatment with PBS, 5 μM TFP, or 4 Gy radiation for 48 h, or pretreated with 5 μM TFP for 24 h combined with 4 Gy radiation and TFP for another 24 h. **d** Caspase 3/7 activation of GBM cells in four treatment groups. **P < 0.01; ***P < 0.001; size bars = 50 μm

We next quantified γ-H2AX foci at different time points after radiation treatment or TFP combined with radiation using immunofluorescence. Radiation induced more γ-H2AX foci after 2 h of treatment. This was reduced considerably from 6 h and onwards. However, TFP treatment resulted in a significant prolongation of the γ-H2AX signal at least 24 h post irradiation in U251 and U87 cells (27% and 21.6%, respectively) compared with radiation alone (10%, *P* < 0.01; 2.3%, *P* < 0.05. Figure 4a, b). Radiation induces apoptosis mainly by generating DSBs. DSBs can be repaired by either non-homologous end joining (NHEJ) or homologous recombination (HR). In NHEJ, ligation occurs regardless of whether the ends come from the same chromosome. Thus loss of genetic information and translocations might occur [25]. HR uses the information that is contained in genetically identical, or almost identical, DNA molecules (usually the sister chromatid) to repair damaged DNA, and therefore, has a higher accuracy of maintaining DNA integrity [26]. The DNA repair protein Rad51 polymerizes onto resected DNA ends to form a nucleoprotein filament and promotes strand exchange between homologous DNA duplexes. As such, Rad51 plays a central role in HR and is crucial for the stability of the genome and the normal cell cycle [27]. After treatment of TFP, we found that the expression of Rad51 and the associated DNA repair proteins BRCA1 and BRCA2, decreased, while γ-H2AX increased in a dose-dependent manner (Fig. 5a). In addition, HR efficiency was calculated using a PCR-based HR assay kit. We found that after TFP treatment, the HR efficiency decreased significantly (*P* < 0.05) compared to the control group (Fig. 5b). TFP might therefore lead to an increase in DSBs and radiosensitivity by inhibiting the expression of central DNA repair proteins.

### Down-regulating cathepsin L might contribute to the radiosensitivity effect of TFP

**Fig. 6:**
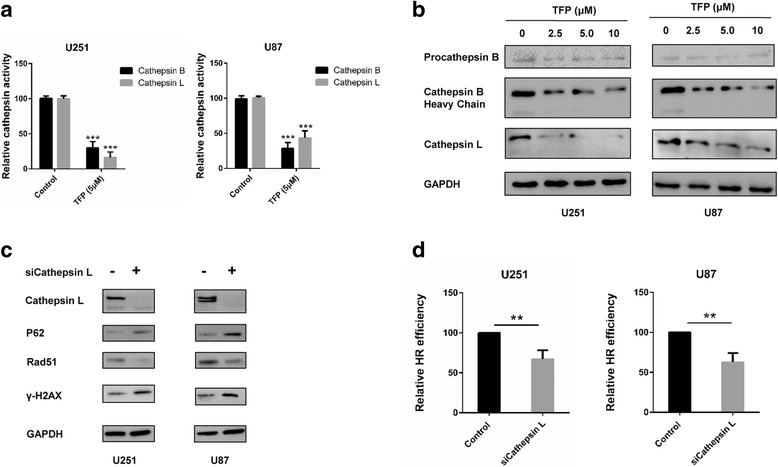
Cathepsin L links autophagy and DNA damage repair to the radiosensitivity effect of TFP. **a** Activity of cathepsin B and L after treatment with PBS (control) and 5 μM TFP. **b** Western blot analysis of cathepsin B heavy chain, procathepsin B and cathepsin L after different TFP treatment doses (0-10 μM). **c** Western blots for cathepsin L, P62, Rad51 and γ-H2AX in U251 and U87 cells before and after cathepsin L knock-down. **d** HR activity of U251 and U87 before and after cathepsin L knock down. **P < 0.01; ***P < 0.001

Lysosomes play an important role during autophagy and among all the lysosomal proteases, cathepsins have multiple roles, not only in the degradation of nonfunctional organelles, but also in the process of tumorigenesis [28]. Earlier studies have also shown that proteasomes as well as lysosomes can contribute to cellular tolerance to various proteotoxic stressors, and confer resistance to chemo-, radio- and immunotherapy [29]. We therefore wanted to assess a putative role of lysosomes as a link between autophagy and DNA damage repair following TFP treatment. Moreover, impaired acidification of lysosomes might affect the activity of cathepsins, which is optimized for low pH [30]. We first examined the enzymatic activities of two main cathepsins (B and L), in response to TFP. With a fluorometric-based kit, we found that after TFP treatment, the activity of cathepsin B and L decreased significantly (Fig. 6a). By western blot analysis, we also found that cathepsin B heavy chain (the mature form of cathepsin), procathepsin B and cathepsin L decreased in a dose-dependent manner (Fig. 6b). Previous studies have shown that cathepsin L is not only involved in autophagy [31–33], but also in radioresistance [34, 35]. After knocking down cathepsin L, we found that the expression of P62 and γ-H2AX increased, while Rad51 decreased (Fig. 6c), which was in consistent with the results observed after TFP treatment. Finally, HR efficiency also decreased significantly after knocking down cathepsin L (Fig. 6d). In conclusion, these data suggest that down-regulation of cathepsin L might explain the radiosensitivity effect of TFP.

### Combining TFP and radiotherapy treatment enhances survival

**Fig. 7:**
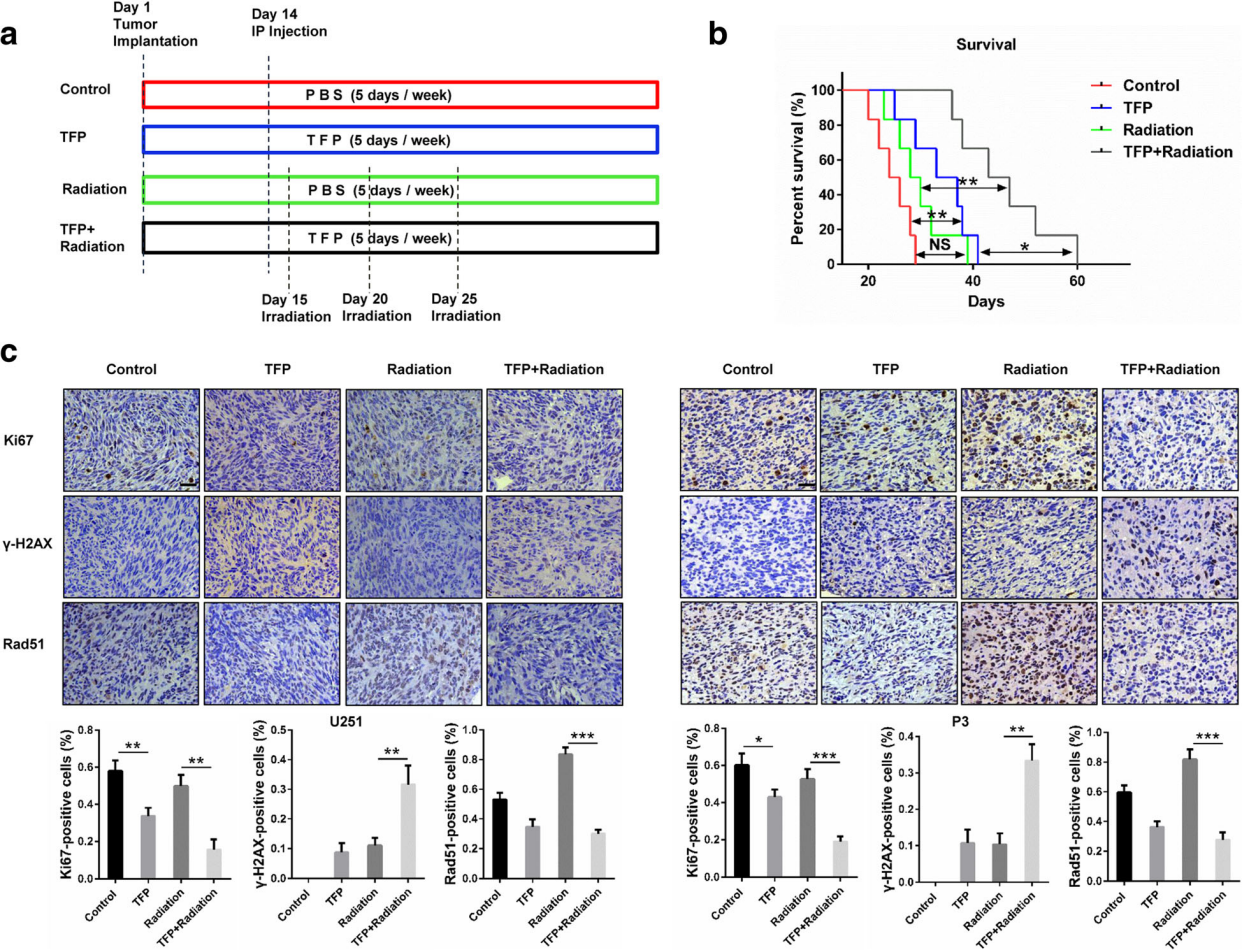
TFP and radiation combination therapy significantly reduces tumor volume and prolongs survival in mice implanted with U251 and P3. **a** Schematic overview of the four different treatment groups in vivo. **b** Kaplan-Meier survival curves in nude mice bearing P3 intracranial xenograft treated with TFP, radiation or combined therapy. **c** Immunostaining for Ki67, γ-H2AX and Rad51 in the paraffin sections from brains from each of the four treatment groups. IP = intraperitoneally; NS = difference not significant (*p* > 0.05); *P < 0.05; **P < 0.01; ***P < 0.001; size bars = 50 μm

To evaluate the antitumor effect of TFP in combination with radiation in vivo, we established orthotopic xenograft models with U251 and P3 cells (Fig. 7a). The survival curves demonstrated no survival benefit from radiation treatment alone (median survival 24.8 and 29.7 days, −control vs radiation respectively, *P* > 0.05), while TFP significantly prolonged survival (median survival 24.8 days vs 33.8 days, control vs TFP respectively). Combined treatment with TFP and radiation significantly increased the overall median survival of animals and produced some long-term survivors (median survival 46 days vs 29.7 days, radiation vs combined treatment, *P* < 0.01; median survival 46 days vs 33.8 days, TFP vs combined treatment, *P* < 0.05. Fig. 7b). Analyses of Ki67 positive cells by immunohistochemistry from TFP treated sections and the combination group showed a dramatic decrease in tumor cell proliferation compared to the control and radiation group (Fig. 7c). Sections stained for γ-H2AX and Rad51 showed that in the TFP treatment group, the expression of Rad51 decreased compared with controls while in the radiation treatment group it was increased. In the combined treatment group, a significantly reduced fraction of cells was positive for Rad51 compared to controls. Animals treated with TFP and radiation displayed a significant increase in γ-H2AX staining compared with either treatment alone (Fig. 7c). In summary, our data demonstrates that TFP sufficiently penetrates the BBB and increases radiosensitivity in vivo.

## Discussion

Recent research has shown that autophagy has a cytoprotective effect during anticancer therapy with DNA-damaging agents. It has also been shown that autophagy inhibition can sensitize tumor cells to such agents [8, 36]. In general, the cytoprotective function of autophagy appears to be linked to apoptosis inhibition through a cross-talk between autophagy and apoptosis regulatory pathways [4]. Therefore, clinical trials have been initiated combining autophagy inhibitors with traditional radio-chemotherapy for the treatment of GBMs [9–11]. However, due to a limited BBB penetration, many autophagy inhibitors do not reach effective concentrations within the brain, −or the dosage is so high that it causes serious side effects. Therefore, novel autophagy inhibitors that can effectively enter the brain are urgently needed.

Drug repurposing has gained increased attention since these drugs have documented safety profiles from clinical use. It is well known that established drugs may have other mechanisms of action beyond the purpose for which they were developed. Drug repositioning also overcome, to a large extent, problems related to laborious and expensive drug development processes [37]. Furthermore, the development of many compounds runs into issues related to safety. Although known as an autophagy inducer [38, 39], an increasing number of studies have illustrated that TFP shows therapeutic efficacy towards various neoplasms, such as lung cancer, malignant peripheral nerve sheath tumors, and leukemia [14–16]. Here, we show that TFP inhibits GBM growth in vitro and in vivo. Mechanistically we show that TFP interrupts autophagy flux by inhibiting the acidification of lysosomes.

Radiation therapy combined with temozolomide (TMZ) treatment represents the standard treatment of care for GBM patients. However, therapeutic efficacy is significantly limited by the development of tumor resistance mechanisms, both towards radiation therapy and TMZ. Radioresistance can develop by multiple factors mediated by the tumor cells as well as by the microenvironment [40]. DNA damage repair is an essential mechanism that is triggered in cells for maintaining genetic integrity, −in order to survive potentially lethal levels of DNA damage. Among all the factors involved in DNA damage repair, HR plays the most prominent role. Early studies have shown that multiple immortalized, tumor cell lines and primary tumor samples overexpress Rad51 [41, 42]. These studies have implicated Rad51 in oncogenesis and indicate that tumor cells show extreme capabilities to repair DNA damage caused by chemotherapy and radiotherapy. In the present work, we show that TFP decreases the expression of the HR proteins Rad51, BRCA1 and BRCA2, followed by increased DNA damage. This indicates that HR efficiency is attenuated during TFP treatment.

In our study, we find that TFP impairs lysosome acidification. Lysosomal proteases, especially cathepsins play an important role in late stage of autophagy [28, 32]. Some studies also demonstrate that cathepsin L contributes to the radioresistance of GBM cells [34, 35]. We show here that the activity as well as protein levels of cathepsin L decreases significantly after TFP treatment. Moreover, western blot analysis shows that cathepsin L silencing increases P62 and γ-H2AX, whereas Rad51 is decreased. Also, knock-down of cathepsin L led to a decreased HR efficiency. These results suggested that TFP might achieve a radiosensitivity effect by down-regulating cathepsin L. Therefore, cathepsin L might be an important factor in the regulation of autophagy, DSBs, and DNA damage repair, which makes it an attractive target in the radiosensitization of GBM.

The BBB is a major impediment to the entry of many drugs into the brain, partly because drugs that are P-glycoprotein (P-gp) substrates are extruded from the brain by the BBB [43]. Studies have shown that TFP inhibits the expression of P-gp [44], which may lead to effective drug concentrations within the brain and also GBMs.

In addition, TFP is also indicated for use in agitation, and in patients with behavioral problems as well as severe anxiety, severe nausea and vomiting, which may improve patients’ symptoms after surgery and radiation therapy. Thus, TFP might provide GBM patients with a better life quality during treatment. Given the long clinical use of TFP in psychotic and non-psychotic patients since the late 1950s, we provide a strong rationale for using TFP for the treatment of GBM patients together with standard therapy.

## Conclusion

In conclusion, the present study revealed a new potential mechanistic insight into the radiosensitization induced by TFP. In our system, TFP treatment effectively decreased the expression of Rad51, BRCA1, BRCA2 and HR. Also, combining TFP and radiation resulted in a significant antitumor effect in orthotopic GBM xenograft models in vivo*.* This study therefore provides the basis for clinical studies combining TFP and radiation.

## Abbreviations

AVs: Autophagic vacuoles
BAF: Bafilomycin A1
BBB: Blood-brain-barrier
CCK-8: Cell counting kit-8
DSBs: Double strand breaks
GBM: Glioblastoma
HR: Homologous recombination
IP: Intraperitoneally
NHEJ: Non-homologous end joining
OTM: Olive tail moment
P-gp: P-glycoprotein
TEM: Transmission electron microscopy
TFP: Trifluoperazine
TMZ: Temozolomide
